# BRCA1 founder mutations compared to ovarian cancer in Belarus

**DOI:** 10.1007/s10689-014-9721-8

**Published:** 2014-04-27

**Authors:** Alena Savanevich, Oleg Oszurek, Jan Lubiński, Cezary Cybulski, Tadeusz Dębniak, Steven A. Narod, Jacek Gronwald

**Affiliations:** 1 Department of Obsetetrics and Gynecology, Grodno State Medical University, Grodno, Belarus; 2 Department of Genetics and Pathology, International Hereditary Cancer Center, Pomeranian Medical University, ul. Połabska 4, 70-115 Szczecin, Poland; 3 Women’s College Research Institute, Toronto, ON Canada

**Keywords:** BRCA1 founder mutation, Ovarian cancer, Belarus

## Abstract

In Belarus and other Slavic countries, founder mutations in the BRCA1 gene are responsible for a significant proportion of breast cancer cases, but the data on contribution of these mutations to ovarian cancers are limited. To estimate the proportion of ovarian cancers in Belarus, which are dependent on BRCA1 Slavic founder mutations, we sought the presence of three most frequent mutations (BRCA1: 5382insC, C61G and, 4153delA) in 158 consecutive unselected cases of ovarian cancer. One of the three founder mutations was present in 25 of 158 unselected cases of ovarian cancer (15.8 %). We recommend that all cases of ovarian cancer in Belarus be offered genetic testing for these founder mutations. Furthermore, genetic testing of the Belarusian population will provide the opportunity to prevent a significant proportion of ovarian cancer.

## Introduction

We and others have previously shown that three recurrent mutations in BRCA1 are founder mutations in the population of Belarus and neighbor countries [1–9]. In aggregate, the three mutations are responsible for approximately 8 % of all breast cancer cases diagnosed in the country [2]. Based on this high prevalence, we recommended that all women with breast cancer in Belarus be tested for the founder mutations. BRCA1 mutations also predispose to ovarian cancer. Recently, Bogdanova and colleagues estimated that 26 % of 201 unselected cases of ovarian cancer from one hospital in Minsk carried one of these founder mutations [3]. This is among the highest proportions of hereditary ovarian cancer reported to date therefore it is important that the finding be confirmed. The aim of this study was to estimate the frequency of the three BRCA1 founder mutations in unselected series of ovarian cancer patients from Belarus. These data have potentially important implications for genetic counseling and testing in Eastern Europe.

## Materials and methods

Ovarian cancer cases were identified from patients treated at clinical base of the Grodno State Medical University in Grodno Regional Clinical Hospital, in October 2008 and May 2011. All patients were inhabitants of the western region of Belarus. The study group consisted of 158 consecutive, newly diagnosed cases of ovarian cancer after surgical treatment, unselected for age or family history. The mean age of diagnosis was 56.2 years (range 27–81 years). The reference pathologist reviewed a representative slide from each cancer to confirm the diagnosis. A family history of breast and ovarian cancer was obtained through questionnaire. Each patient provided written informed consent to take part in the study. The study was approved by the ethics committees of the Pomeranian Medical University in Szczecin, and of the Grodno State Medical University.

DNA was extracted from peripheral blood lymphocytes using the Puregene kit (Gentra) according to the manufacturer’s instructions. Two BRCA1 mutations (4153delA and 5382insC) were studied using ASA-PCR and one mutation (C61G) was detected with RFLP-PCR, as described elsewhere [10].

## Results

A BRCA1 mutation was detected in 25 of the 158 (15.8 %) unselected ovarian cancer cases. The 5382insC mutation was the most common—it was diagnosed in fifteen patients, followed by the 4153delA mutation in eight patients and the C61G mutation in two patients. The median age of diagnosis of the 25 hereditary ovarian cancers was 53.9 years (range 41–79 years), compared with a median age of diagnosis of 56.6 years (range 27–81 years) for the 133 cases without a mutation. A mutation was found in 22.4 % of women diagnosed with ovarian cancer at or under the age of 50 compared to 12 % of women diagnosed at a later age. Among the 25 women with ovarian cancer and a BRCA1 mutation, only eight reported a first- or second-degree relative with breast or ovarian cancer (32 %). A mutation was present in 30.8 % of ovarian cancer patients with a positive family history and in 12.9 % of women with a negative family history (Table 1).

**Table 1:** Prevalence of BRCA1 mutations in ovarian cancer by age of onset and family history

	Number of cases	Number with a mutation	Proportion with a mutation (%)
Age group			
27–50	58	13	22.4
51+	100	12	12
Family history			
Positive	26	8	30.8
Negative	132	17	12.9
All cases togother	158	25	15.8

## Discussion

Belarus is a former Soviet republic of 10 million inhabitants that is bordered by Poland, Lithuania, Latvia, Russia and Ukraine. We found that 16 % of unselected cases of ovarian cancer in Belarus carried one of three founder mutations in the BRCA1 gene. This is significant higher than in Canada or Poland [11, 12]. A reported family history was a strong predictor of the presence of a mutation, as was a young age of onset. More than half of mutations (13 of 25) occurred among women diagnosed below the age of 50. It should be noted that in this series, similarly to earlier study [3], majority of mutation-positive patients did not have a positive family history of breast or ovarian cancer.

This is the second study of BRCA1 mutations in ovarian cancer patients from Belarus. Our patients originated from the region of Grodno in the western region of Belarus. Bogdanova and colleagues reported a prevalence of mutations of 26 % for ovarian cancer patients from the region of Minsk [3]. It is possible that there are regional differences in the distribution of mutations within the country, however, the relative distribution of the three mutations in the two regions was roughly similar.

In both studies, the 5382insC mutation was the most common and in both studies the C61G mutation was relatively rare. The 5382insC mutation is the most common mutation in Poland, Russia and Belarus [1–6]. 5382insC is also a founder mutation in other Slavic countries, such as the Czech Republic [13] Slovenia [14] and northern Greece [15] and is the second most common BRCA1 mutation in the Ashkenazi Jewish population [16, 17].

There are several limitations to our study. The number of cases is relatively small and the results are based on 25 mutation-positive ovarian cancer cases. We screened for only the three founder mutations and it is possible that other non-founder mutations were missed. Nevertheless, we believe that it is reasonable and cost effective to offer to test all breast and ovarian cancer patients in Belarus for the three founder mutations. A high proportion of carriers of BRCA1 mutations do not have a strong family history of either type of cancer and it is therefore unreliable to rely on family history in order to decide upon whom to test. Preventive bilateral salpingo-oophorectomy is effective method to reduce ovarian cancer incidence in BRCA1 carriers, improve survival and should be advocated after 35 years of age when childbirth is completed [18]. It can be widely used in Belarusian BRCA1 carriers to prevent significant number of ovarian and breast cancers.
